# Bow Hunter’s Stroke Caused by Cryptic Recanalization of an Occluded Vertebral Artery: A Case Report

**DOI:** 10.7759/cureus.73246

**Published:** 2024-11-07

**Authors:** Junpei Nagasawa, Ayano Matsuoka, Makiko Ogawa, Mari Shibukawa, Osamu Kano

**Affiliations:** 1 Neurology, Toho University Faculty of Medicine, Tokyo, JPN

**Keywords:** acute ischemic stroke (ais), bow hunter's syndrome, embolic stroke of undetermined source, vertebral arteries, vertebrobasilar stroke

## Abstract

An 80-year-old man was admitted to our hospital with acute cerebellar infarction. Conventional magnetic resonance angiography and computed tomography angiography (CTA) showed occlusion of the right vertebral artery (VA). Carotid ultrasonography revealed that the right VA was narrowed at its entry point into the transverse foramen near C6. Given the location of the VA stenosis, the blockage may have been due to compression from the cervical spine. Therefore, we assessed the right VA blood flow by moving the neck. The cervical spine was rotated left and right for evaluation; however, no blood flow was observed. The neck was then flexed 30° from the neutral position, and blood flow in the VA was confirmed using color and pulse Doppler. Similarly, CTA and cerebral angiography confirmed that the right VA, which was occluded in the neutral neck position, recanalized when the neck was flexed. We hypothesized that a thrombus had formed in the right VA during occlusion. When the neck was flexed, the right VA reopened, allowing the thrombus to move, resulting in embolic cerebral infarction. In typical bow hunter's syndrome (BHS), VA occlusion occurs in the neutral position, and blockage happens during rotation. In this case, the VA was blocked in the neutral position and recanalized during flexion. This so-called "hidden BHS," as seen in this case, is easily overlooked, highlighting the importance of careful evaluation.

## Introduction

Bow hunter's syndrome (BHS) was first reported in 1972 by Sorensen [1]. BHS involves vertebral artery (VA) compression due to head and neck rotation or extension, leading to dizziness, syncope, and potentially cerebral infarction [2,3]. BHS is an important differential diagnosis for posterior circulation cerebral infarction.

In typical BHS, the VA remains patent in the neutral position but becomes compressed during neck movement. However, we report a case in which the VA, occluded in the neutral neck position, recanalized during neck movement, leading to cerebral infarction. Such cases of "hidden BHS" are rare and easily overlooked, so care must be taken not to miss this condition.

## Case presentation

**Figure 1:**
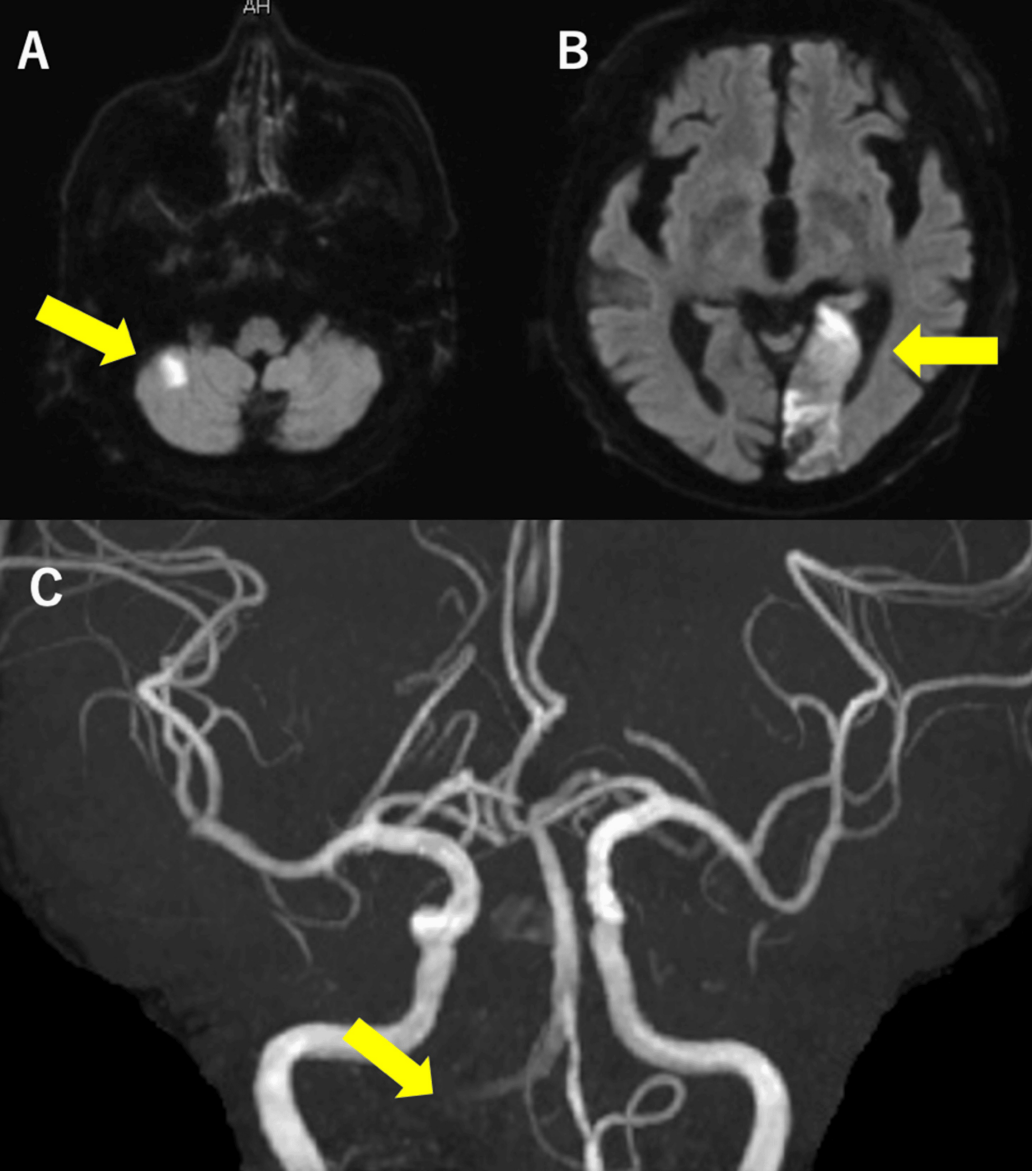
Brain MRI at the onset of cerebral infarction MRI/DWI shows acute cerebral infarction in the right cerebellar hemisphere (A, arrow) and left occipital lobe (B, arrow). MRA shows poor visualization of the right VA (C, arrow). MRI: magnetic resonance imaging, DWI: diffusion-weighted imaging, MRA: magnetic resonance angiography

**Figure 2:**
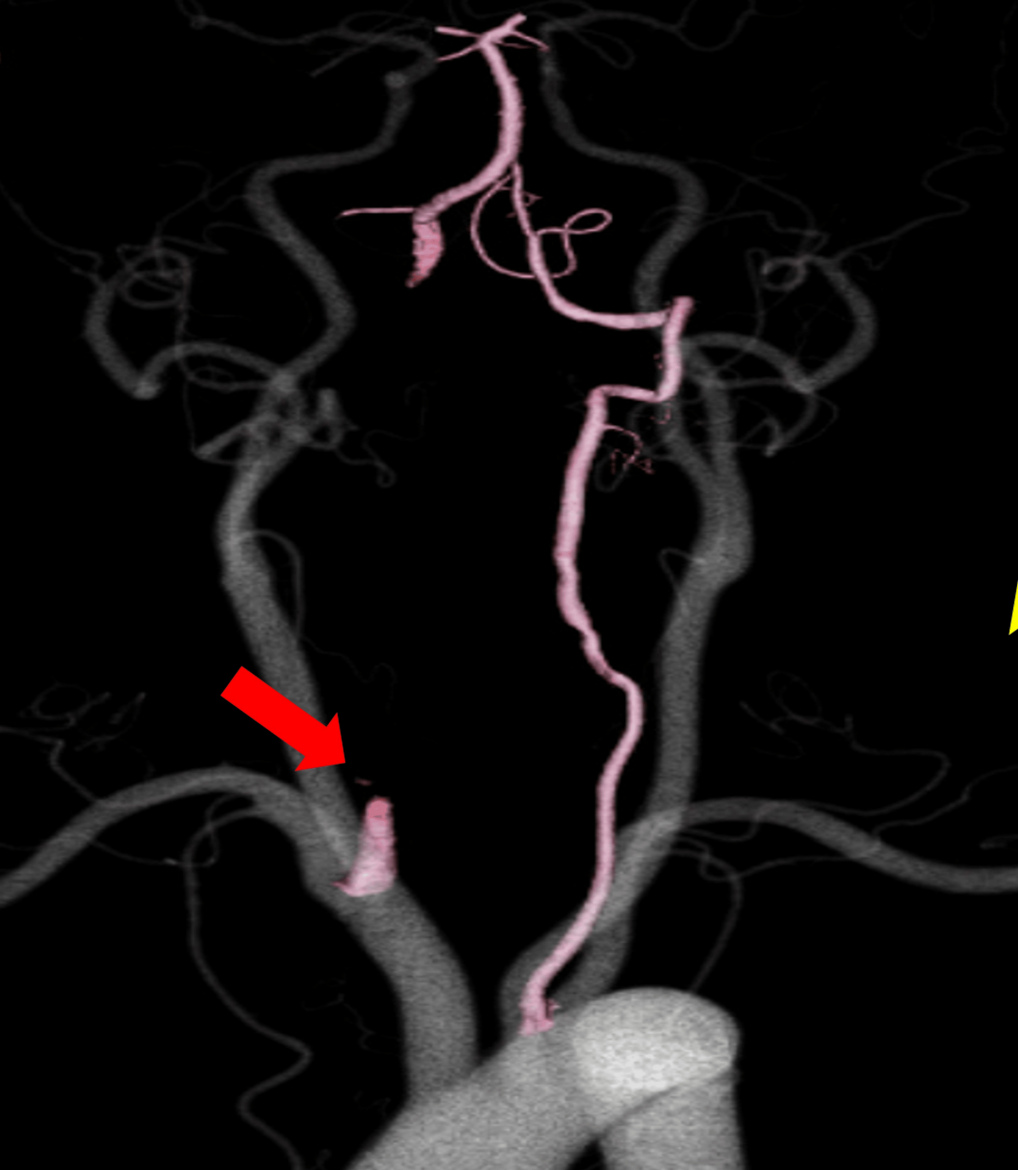
CTA at the onset of cerebral infarction CTA shows occlusion of the right VA in the cervical portion (arrow). CTA: computed tomography angiography, VA: vertebral artery

An 80-year-old man with a history of diabetes experienced unsteadiness while walking. After five days, his dizziness worsened, and he developed nausea and right hemianopsia. He was admitted to our hospital for emergency treatment. His vital signs were stable, and his Glasgow Coma Scale (GCS) score was E4V5M6. Neurological examination revealed ataxia in the right upper and lower extremities, as well as right homonymous hemianopsia. His National Institutes of Health Stroke Scale (NIHSS) score was 4. Laboratory test results were unremarkable. Brain magnetic resonance imaging (MRI) and diffusion-weighted imaging (DWI) showed acute cerebral infarction in the cerebellar hemispheres and the left occipital lobe (Figure 1A-1B). Brain magnetic resonance angiography (MRA) revealed poor visualization of the right VA (Figure 1C), and head and neck computed tomography angiography (CTA) suggested occlusion of the right VA (Figure 2). The patient was hospitalized, and oral aspirin was started.

**Figure 3:**
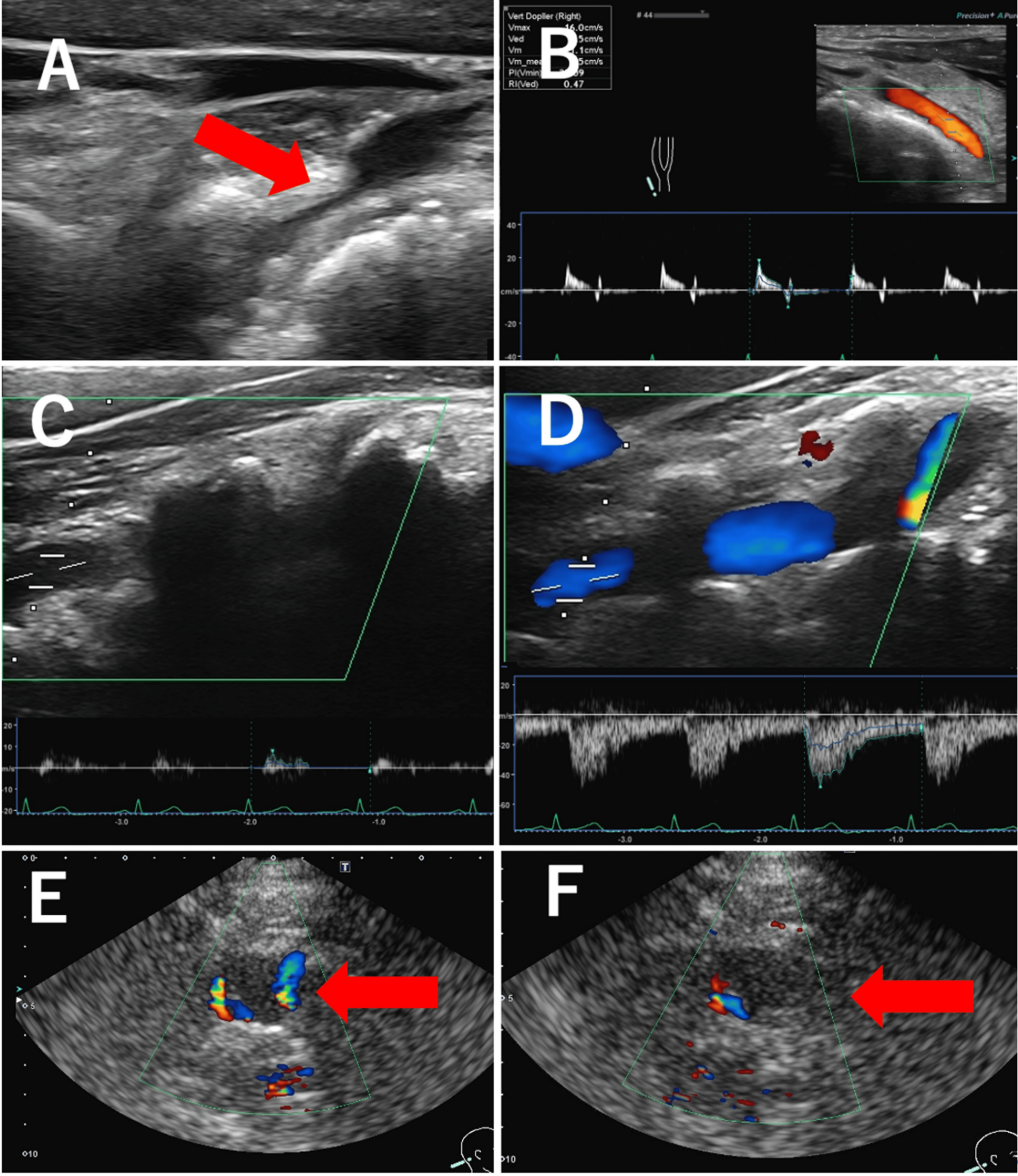
Carotid ultrasound Carotid ultrasound shows that the right VA was narrowed at the point where it entered the transverse foramen near C6 (A, arrow). Doppler waveforms in the VA proximal to the narrowed area showed no diastolic blood flow, indicating distal occlusion (B). The cervical spine was rotated left and right for evaluation, but blood flow was still not observed (C). The neck was flexed 30º from the neutral position, and blood flow in the VA was confirmed using color Doppler and pulse Doppler (D). In TC-CFI, blood flow in the intracranial right VA could not be confirmed in the neutral position (F, arrow) or when rotated left and right but could be confirmed when the neck was flexed (E, arrow). VA: vertebral artery, TC-CFI: transcranial color-flow imaging

Carotid ultrasonography revealed a narrowing of the right VA at the point where it entered the transverse foramen near C6 (Figure 3A). Doppler waveforms of the VA proximal to the narrowed area showed no diastolic blood flow, indicating distal occlusion (Figure 3B). Given the location of the VA stenosis, the blockage may have been due to compression of the cervical spine. To evaluate blood flow in the right VA, we moved the neck. The cervical spine was rotated left and right for assessment; however, no blood flow was observed (Figure 3C). When the neck was flexed 30° from the neutral position, blood flow in the VA was confirmed using color and pulse Doppler (Figure 3D). In transcranial color-flow imaging (TC-CFI), blood flow in the intracranial right VA could not be confirmed in the neutral position (Figure 3F) or when rotated left and right, but it was confirmed when the neck was flexed (Figure 3E).

**Figure 4:**
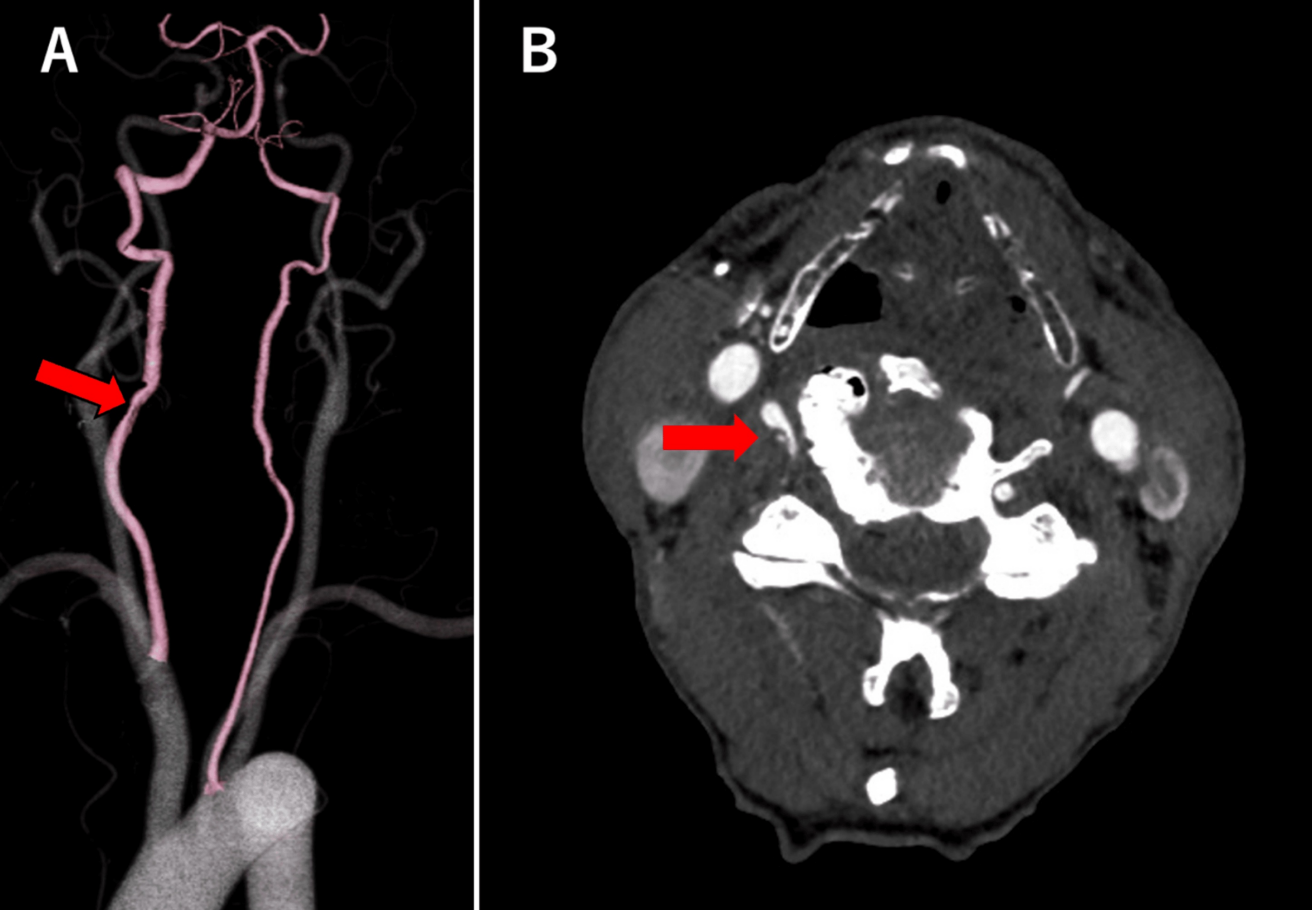
CTA performed with the neck in the flexed position CTA performed with the neck in the flexed position showed that recanalization of the right VA (A; arrow) narrowed at the site where it entered the cervical transverse foramen (A, B, arrow). CTA: computed tomography angiography, VA: vertebral artery

CTA performed with the neck flexed showed recanalization of the right VA (Figure 4A-4A), which had been blocked in the neutral neck position on the previous CTA and narrowed at the point where it entered the cervical transverse foramen (Figure 4A-4B).

**Figure 5:**
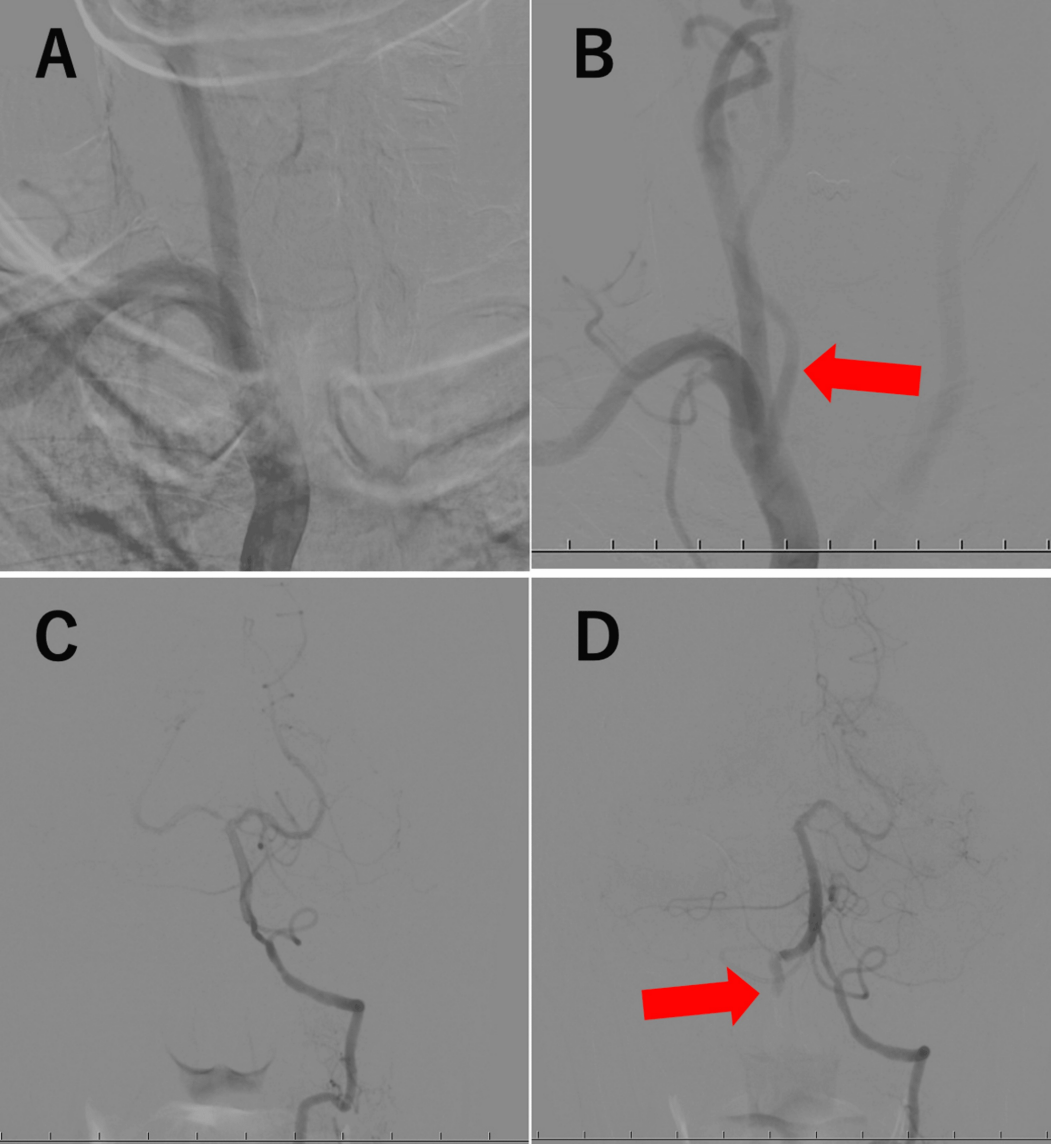
Cerebral angiography In cerebral angiography, angiography from the right brachiocephalic artery did not reveal the right VA when the neck was in the neutral position or rotated left and right (A), but blood flow in the right VA was observed when the neck was flexed (B, arrow). Left VA imaging showed only antegrade blood flow when the neck was flexed in the neutral position (C), but reflux into the right VA and visualization of the right PICA were confirmed when the neck was in the neutral position (D, arrow). VA: vertebral artery, PICA: posterior inferior cerebellar artery

Angiography of the right brachiocephalic artery did not reveal the right VA when the neck was in the neutral position or rotated to the left or right (Figure 5A); however, blood flow in the right VA was observed when the neck was flexed (Figure 5B). Imaging of the left VA showed only antegrade blood flow with the neck flexed in the neutral position (Figure 5C); however, reflux into the right VA and visualization of the right posterior inferior cerebellar artery were confirmed when the neck was in the neutral position (Figure 5D).

Accordingly, the right VA was occluded in the neutral position of the neck, but blood flow in the posterior circulation system was compensated by the left VA. However, a thrombus formed in the right VA at the time of occlusion. When the neck was flexed, the right VA reopened, allowing the thrombus to flow, which caused an embolic cerebral infarction. Therefore, prasugrel was added to aspirin for dual antiplatelet therapy. Furthermore, we considered surgical procedures, such as decompression and fixation of the cervical spine or parent-artery embolization.

**Figure 6:**
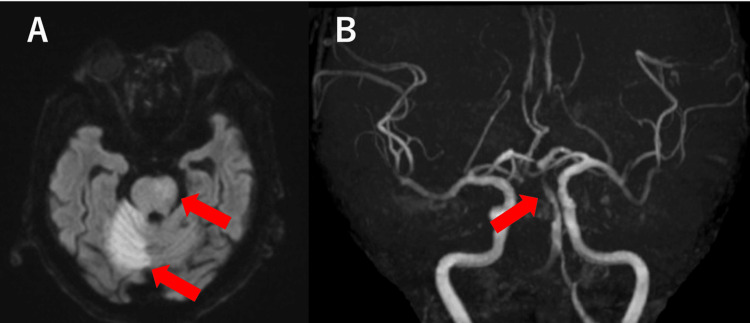
Brain MRI at the time of recurrent cerebral infarction Brain MRI revealed a new right cerebellar hemisphere infarction and a left pontine infarction (A, arrow). MRA showed slightly poor visualization of the basilar artery, including its top (B, arrow). MRI: magnetic resonance imaging, MRA: magnetic resonance angiography

However, 28 days after admission, before a surgical procedure was decided upon, the patient experienced impaired consciousness (GCS score: E2V2M4) and paralysis of the right upper and lower limbs upon awakening. The NIHSS score was 26. Brain MRI revealed a new infarction in the right cerebellar hemisphere and left pontine region (Figure 6A). MRA showed slightly poor visualization of the basilar artery, including its top (Figure 6B).

**Figure 7:**
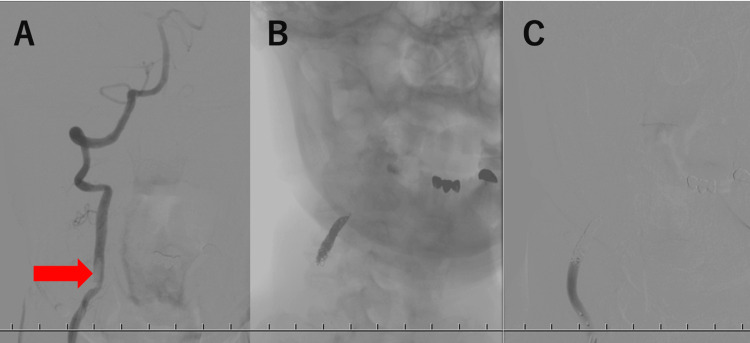
Emergency angiography for recurrent cerebral infarction A right VA angiography with the patient in a neck-flexed position revealed a large thrombus distal to the stenosis (A, arrow). Fifteen coils were used (Figure B), and subsequent angiography confirmed sufficient occlusion of the right VA (Figure C). VA: vertebral artery

Emergency cerebral angiography was performed, and the right VA angiography with the patient in a flexed neck position revealed a large thrombus distal to the stenosis (Figure 7A). To prevent thrombus embolization, an emergency mechanical thrombectomy was conducted. A Trevo NXT 4 mm × 28 mm (Stryker Corp., Kalamazoo, MI, USA) was deployed at the thrombus site in the right VA using a Trevo Track 21 microcatheter (Stryker Corp., Kalamazoo, MI, USA). A blood clot approximately 4 mm in size was retrieved from the stent. Post-procedure right VA angiography revealed no signs of a thrombus.

To prevent the recurrence of the cerebral infarction, occlusion of the right VA parent vessel was performed. A 6 Fr guiding catheter was placed in the right VA, and an Excelsior SL-10 stent (Stryker Corp., Kalamazoo, MI, USA) was positioned immediately proximal to the stenosis. A Target 360 coil, 5 mm (Stryker Corp., Kalamazoo, MI, USA), was inserted to form a cage, followed by additional coils of progressively smaller sizes. A total of 15 coils were used (Figure 7B), and subsequent angiography confirmed sufficient occlusion of the right VA (Figure 7C). No new neurological symptoms were observed following the endovascular treatment.

There was no recurrence of the cerebral infarction, and the patient underwent rehabilitation. Fifty-nine days after admission, the patient was transferred to a nursing hospital with a modified Rankin scale score of 5.

## Discussion

BHS, also known as rotational VA occlusion syndrome, is a condition that causes VA compression due to head and neck rotation or extension, resulting in dizziness and syncope, and may lead to cerebral infarction [2,3].

The mechanisms by which BHS causes cerebral infarction have been reported to include hemodynamic insufficiency due to occlusion or severe stenosis of the compressed VA and embolic mechanisms resulting from thrombus formation due to damage to the vascular endothelium [4]. Regarding the site of VA compression by the bone, Jost and Dailey summarized the data of 126 patients with BHS and pointed out that the occlusion was mostly located between C3 and C7 (58%), followed by C1-C2 (36%), with only 6% occurring proximal to C7 or distal to C1 [5].

In normal BHS, the VA is patent when the cervical spine is in a neutral position and becomes blocked during rotation. In this case, however, the VA was blocked in the neutral neck position and recanalized during flexion. Because the VA was occluded with the patient in a neutral position, which is the usual examination position, it was difficult to distinguish this from chronic occlusion using conventional MRA, CTA, and carotid artery ultrasound examinations.

Mori et al. reported a similar case in which the VA was blocked in the neutral position of the neck and recanalized during rotation, causing cerebral infarction, and termed this "hidden BHS (HBHS)" [6]. Miyamoto et al. summarized six cases of HBHS, including one of their own and five cases identified in a literature review [7]. The authors reported that, compared with normal BHS, hidden BHS is more likely to involve a mechanism in which a thrombus formed during occlusion causes embolic cerebral infarction during recanalization rather than a direct perfusion failure due to VA occlusion. Therefore, antiplatelet agents are more likely to cause recurrence, and the possibility that anticoagulant therapy is effective should be considered. Consistent with this report, in the present case, thrombus formation at the time of occlusion was believed to be the cause of cerebral infarction, which recurred despite antiplatelet therapy.

There is no established standard treatment for HBHS; however, it is appropriate to consider it the same as normal BHS. Treatment for normal BHS includes conservative antithrombotic therapy and cervical immobilization, as well as surgical treatments such as decompression, fixation of the cervical spine, and parent artery embolization. The reported treatments for HBHS include cervical decompression [7-9] and parent artery embolization [6].

A unique feature of this case was that, whereas in all previous cases of HBHS, the VA was recanalized by neck rotation, in this case, it was recanalized by neck flexion. The movements that trigger a normal BHS include not only neck rotation but also flexion and extension. The exact proportion of patients with a normal BHS involving neck flexion or extension remains unknown. In a literature review of normal BHS [5], of 114 cases in which a trigger was described, 102 (89%) were triggered by neck rotation, 10 (8%) by rotation plus extension, and two (1%) by extension alone. As described above, even in normal BHS, cases in which neck extension alone is the trigger are rare, and this is even rarer in cases of hidden BHS. We found five previous cases of hidden BHS [6-10], and in all cases, the VA was recanalized when the neck was rotated to the contralateral side. Therefore, this is the only case in our review in which the VA was recanalized with cervical flexion in a patient with hidden BHS. HBHS is easy to overlook; therefore, hidden BHS must be carefully managed, and its evaluation requires not only neck rotation but also neck flexion and extension.

## Conclusions

Here, we report a case of BHS stroke caused by cryptic recanalization of an occluded VA, in which the VA recanalized only during neck flexion. Cervical ultrasound and cerebral angiography are useful for diagnosing HBHS; however, it is necessary to rotate, flex, and extend the neck during these procedures. There is a high possibility of recurrence with standard medical treatment alone; therefore, care must be taken to avoid overlooking this condition.
